# Breast Cancer Treatments: Drugs Targeting the PI3K/AKT/mTOR Pathway, TNBC Therapy and Future Directions: A Review

**DOI:** 10.3390/life15101583

**Published:** 2025-10-10

**Authors:** Klaudia Dynarowicz, Dorota Bartusik-Aebisher, Katarzyna Koszarska, Aleksandra Kotlińska, David Aebisher

**Affiliations:** 1 Department of Biochemistry and General Chemistry, Faculty of Medicine, University of Rzeszów, 35-310 Rzeszów, Poland; kdynarowicz@ur.edu.pl (K.D.); dbartusikaebisher@ur.edu.pl (D.B.-A.); 2 English Division Science Club, Faculty of Medicine, University of Rzeszów, 35-359 Rzeszów, Poland; kk117603@stud.ur.edu.pl (K.K.); ak117604@stud.ur.edu.pl (A.K.); 3 Department of Photomedicine and Physical Chemistry, Faculty of Medicine, University of Rzeszów, 35-359 Rzeszów, Poland

**Keywords:** breast cancer therapy, PI3K/AKT/mTOR pathway, TNBC therapy, nanomedicine, nanoaqualip technology

## Abstract

Breast cancer affects women at an increasingly younger age, with genetic predispositions and other factors contributing to its second-highest cancer mortality rate. The diversity of pharmacological treatment stems from its heterogeneity, which favors a more precise approach to each subtype. Despite the extensive advances in medicine in recent decades, the problem of treating cancer patients remains significant. The problem with modern therapeutic methods is low effectiveness, emerging side effects, difficulty in eliminating all cancer cells, and the quite common use of monotherapy and the associated drug resistance, which may lead to disease progression. The aim of this review is to present the latest therapeutic strategies (combination therapies) used in the treatment of breast cancer. PubMed databases and clinical data from ClinicalTrials.gov were used for this purpose. The review included characteristics of the latest clinical trials from the last year (2024–2025), which present currently recruiting studies of breast cancer treatment with immunotherapy. The review also presented characteristics of clinical trials from the last 5 years (2020–2025) using nanoparticles as an adjunct to breast cancer treatment. Articles published between 2016 and August 2025 (excluding articles that describe the first use of a given drug) were included in the review. The review analyzed drugs targeting molecular targets, including intracellular pathways responsible for cell cycle regulation, as well as new directions such as nanotechnology in treatment breast cancer.

## 1. Introduction

**Figure 1:**
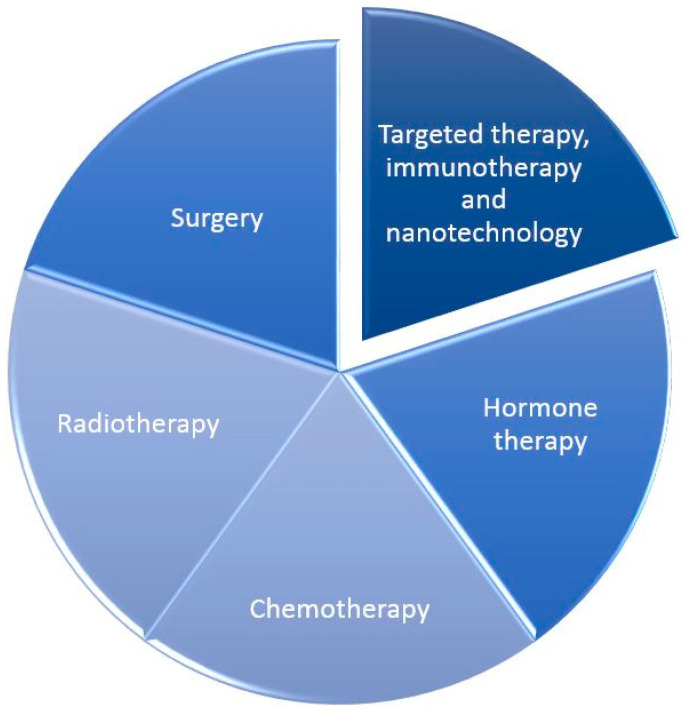
Types of therapeutic methods in the treatment of breast cancer.

Despite extensive technological advances in medical fields such as oncology, radiology, and laboratory diagnostics, cancer is still undoubtedly the leading cause of death worldwide and represents not only a health problem but also a social and demographic one [1,2,3]. According to the 2024 reports of the National Cancer Institute (NCI) Surveillance, Epidemiology, and End Results (SEER) program and the National Program of Cancer Registries (NPCR), breast cancer currently accounts for approximately 32% of all malignancies in women worldwide [4], ranking first in this group. In terms of mortality, it is the second most common cancer, after lung cancer, and accounts for 15% of all cancer deaths (lung cancer accounts for approximately 21%). The mortality rate among all people diagnosed with breast cancer is approximately 15%, and the incidence rate has been slowly increasing by about 0.6% annually since the mid-2000s [4]. There are various therapeutic approaches to treating breast cancer. These depend on the tumor type, the patient’s condition, and the treatment setting [5,6,7]. The most popular methods include surgery, radiotherapy, chemotherapy, and hormone therapy (Figure 1).

Recent advances in understanding the molecular biology of breast cancer have led to the development of targeted therapies and immunotherapies, which aim to exploit the specific genetic and immunological features of different cancer subtypes [8,9]. In recent years, drugs targeting the PI3K/AKT/mTOR pathway have become one of these therapeutic tools [10]. New drugs are being approved by the FDA each year. One of the main sources of knowledge about the current therapeutic status of breast cancer is registered clinical trials. The aim of this review is therefore to characterize the latest drugs targeting the PI3K/AKT/mTOR pathway and to present the characteristics of the latest registered clinical trials using both immunotherapy and nanoparticles. Taking into account the latest treatment trends, progress in breast cancer nanotherapy and Nanoaqualip technology is also presented. The ClinicalTrials.gov database was used to present the characteristics of clinical trials. The following studies using immunotherapy were selected: condition: breast cancer, intervention/treatment: Immune Checkpoint Inhibitors, date range: 1 January 2024–1 January 2025. This review presents those already recruiting patients. The following studies using nanoparticles were selected: condition: breast cancer, intervention/treatment: nanoparticles, study phases: 2 and 3, date range: 1 January 2020–1 August 2025 (a wider range of years was chosen rather than a single year to represent more registered clinical trials). The review presents all eight trials, both those that have not yet started and those currently recruiting patients. This review presents the latest FDA-approved drugs for the treatment of breast cancer in the last two years. Articles cited in the review are from the Pubmed database from the years 2016–2025 (excluding articles that describe the first use of a given drug), selected based on the following keywords: drugs targeting PI3K/AKT/mTOR pathway breast cancer, TNBC therapy, nanoparticles in breast cancer. Full-text articles written in Polish or English, after abstract review, were included in the review.

## 2. Selected Therapeutic Approaches in Breast Cancer

### 2.1. Targeting PI3K/AKT/mTOR Pathway

The Ras/MAPK and PI3K/AKT/mTOR signaling pathways play a key role in cell cycle regulation by affecting cyclin D (mainly D1), which forms a complex with CDK4/6 (serine–threonine kinases) phosphorylates the Rb protein [11,12,13,14]. Then, in this pathway, the Rb-activated transcription factor E2F activates genes necessary for the cell to enter the S phase of the cell cycle. Studies show that mutations in the PI3K/AKT/mTOR pathway are present in even over 70% of breast cancer cases, which is why drugs targeting its elements are widely used, especially in combined treatment in patients with resistance to hormonal therapy, achieving better effects than drugs from these groups used separately [12].

**Figure 2:**
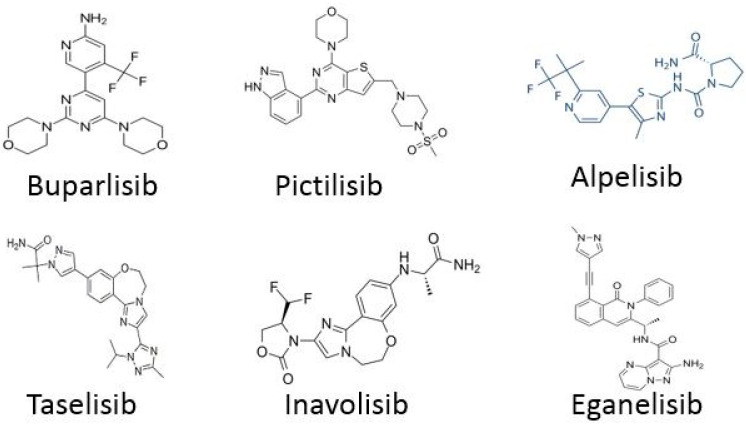
New drugs in clinical trials targeting PI3K pathway.

**Figure 3:**
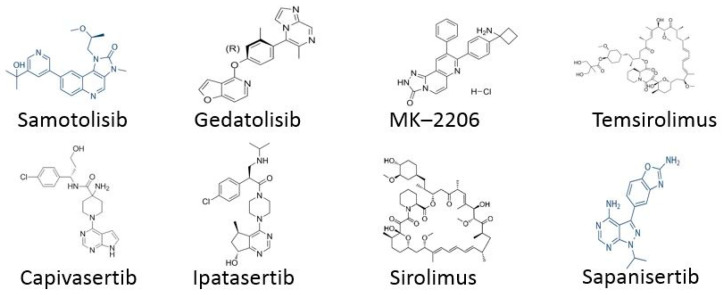
New drugs in clinical trials targeting AKT/mTOR pathway.

Drugs targeting cell cycle control pathways have found wide application in oncology, not only in the treatment of breast cancer but also many others, e.g., lung cancer, pancreatic cancer or melanoma [15,16,17]. New drugs targeting the PI3K pathway in HR+, HER2- breast cancer include Buparlisib, Pictilisib, Alpelisib, Taselisib, and Inavolisib (Figure 2 and Figure 3). Clinical trials are currently evaluating the efficacy of these drugs, with a view to their approval and use on a larger scale. The buparlisib clinical trial was a multicenter, phase III study designed to determine the efficacy and safety of buparlisib plus fulvestrant versus placebo plus fulvestrant [18,19,20,21]. The study population consisted of postmenopausal women with breast cancer (HR-positive, HER2-negative) treated with an aromatase inhibitor. The results clearly demonstrated that median progression-free survival was significantly longer in the buparlisib group compared to placebo. Despite satisfactory results, significant or severe adverse events also occurred, primarily elevated aspartate aminotransferase levels, hyperglycemia, hypertension, and fatigue. It is important to emphasize that these effects occurred in both patients receiving buparlisib and those receiving placebo. Several patients also died, but the cause was metastatic breast cancer, and in one case, heart failure. As the authors present, the efficacy of buparlisib supports the use of PI3K inhibitors in combination with endocrine therapy in breast cancer patients. Pictilisib is used in clinical trials to treat breast tumors with various characteristics [22,23,24,25,26,27]. Registered clinical trials are evaluating the use of pictilisib in combination with fulvestrant in patients with advanced breast cancer whose tumors harbor a PIK3CA mutation. Unfortunately, the results of the clinical trial were unsatisfactory, as the use of pictilisib in combination with fulvestrant did not significantly improve progression-free survival. Furthermore, significant toxicity occurred, significantly reducing the effectiveness of the treatment. Alpelisib is a drug primarily used in the treatment of advanced breast cancer [28,29,30,31,32,33]. Clinical trials demonstrate the combination of alpelisib with fulvestrant or letrozole in patients with HR-positive, HER2-negative breast cancer with PIK3CA mutation (s) in their tumors. Interpretation of the results indicates that the activity of alpelisib with fulvestrant was relatively good with low toxicity. The results are satisfactory, which may lead to expansion of the ongoing study and further verification of the results. Taselisib is a drug used in clinical trials for the treatment of lymphoma, breast cancer, ovarian cancer, solid tumors, and other conditions. In clinical trials, taselisib is administered in combination with fulvestrant [34,35]. The control group is treated with a placebo. The results were more than satisfactory. Positive antitumor effects were observed in patients receiving taselisib–fulvestrant therapy. As the authors state, these results are significant for better understanding the therapeutic impact of this type of combination therapy. These types of clinical trials highlight the importance of combined, targeted therapies [34,35]. Inavolisib is an innovative drug being studied in the treatment of various types of cancer. It belongs to a class of drugs called PI3K inhibitors, which target a specific pathway in cancer cells that promotes their growth and survival [36,37]. In the clinical trial, this drug was administered to patients with locally advanced or metastatic breast cancer with a PIK3CA mutation. The efficacy of inavolisib in combination with palbociclib and fulvestrant was assessed compared to placebo. Median overall survival was higher in patients receiving inavolisib compared to those receiving placebo treatment. Unfortunately, the occurrence of significant side effects forced the authors to discontinue the study and treatment [36,37]. Adverse events observed included hyperglycemia, stomatitis or mucositis, diarrhea, dry eye syndrome, and blurred vision. The current clinical trial is positive, as inavolisib treatment improved overall survival. However, emerging side effects require further research in this area.

For TNBC, a newly analyzed drug is Eganelisib. Eganelisib is a highly selective small-molecule drug with potential immunomodulatory and anticancer effects [38,39]. After administration, eganelisib prevents the activation of signaling pathways, which may lead to reduced cell proliferation in cancer cells. A clinical trial is currently being registered to determine the effectiveness of this drug in patients with TNBC. The results are not yet available, but they may demonstrate promising anticancer activity [38,39].

Newly approved drugs targeting the AKT/mTOR pathway in ER+, HR+, and HER2- breast cancers include Gedatolisib, MK-2206, Capivasertib, Sirolimus, Temsirolimus, Sapanisertib, and Vistusertib. Clinical trials are currently underway to assess the effectiveness of these drugs, with a view to their approval and use on a larger scale.

Gedatolisib is an experimental drug being studied for its potential in treating various types of cancer [40,41,42,43]. This drug belongs to a class of drugs called PI3K/mTOR inhibitors, meaning it works by blocking specific proteins involved in the growth and survival of cancer cells. Clinical trials for Gedatolisib are multicenter, open-label studies in patients with metastatic breast cancer [40,41,42,43]. The proposed clinical trials aim to determine the maximum tolerated dose of gedatolisib with other combination drugs and to estimate the objective response rate. MK2206, a clinically advanced AKT inhibitor, is used to treat patients with breast cancer [44]. Although MK-2206 is not currently being developed further, this class of drugs continues to generate clinical interest. MK-2206 is estimated to have improved survival after treatment completion. It is important to note that the drug itself is characterized by some toxicity. The most significant grade 3–4 toxicity was rash. Tools such as magnetic resonance imaging and molecular characterization of the results are used to analyze the efficacy of MK-2206 administration. Capivasertib specifically targets and inhibits protein kinase B. By blocking the protein, capivasertib aims to slow or stop the growth of cancer cells. The Phase III clinical trial is evaluating the efficacy of capivasertib in combination with fulvestrant versus placebo in combination with fulvestrant in the treatment of patients with HR+/HER2- metastatic breast cancer after relapse or progression [45,46]. The main finding from the clinical trials was that patients who took capivasertib plus fulvestrant lived longer without relapse compared to those treated with placebo plus fulvestrant. It’s worth noting that this result was observed in all participants recruited for the study. This phenomenon was also observed in participants whose tumors had detectable genetic changes in genes called PIK3CA and AKT1. The most common side effects were diarrhea and rash, which the authors say was as expected. Sirolimus, also known by the trade names Rapamune and Rapamycin, is an immunosuppressant drug [47,48]. It was originally approved by the U.S. Food and Drug Administration to prevent kidney transplant rejection. However, researchers are investigating its potential use in treating various other medical conditions. The clinical trials included patients with metastatic breast cancer who were given sirolimus in combination with hormone therapy. Progression-free survival rates in patients treated with sirolimus and everolimus were similar. As in every study, side effects occurred. The most common adverse events were lipid metabolism disorders and stomatitis. However, all recorded side effects were mild. It is worth noting that patients with alterations in the PI3K/AKT/mTOR pathway responded better to sirolimus than those without alterations. Based on the conducted clinical trials, sirolimus is a potentially effective treatment option for patients with hormone receptor-positive advanced breast cancer. Temsirolimus is a derivative and prodrug of sirolimus used in the treatment of renal cell carcinoma. It is an mTOR kinase inhibitor. It is worth noting that temsirolimus is not used in patients with breast cancer. However, clinical trials are being registered that incorporate this drug into the treatment protocol. However, the results are currently unsatisfactory. No overall improvement in the primary endpoint was observed. According to the authors, adding temsirolimus to letrozole did not improve treatment in patients with advanced breast cancer. Further clinical trials may be needed to confirm or refute this finding [49].

Sapanisertib is an experimental mTOR kinase inhibitor used in clinical trials to inhibit the activity of mTORC1 and mTORC2 proteins in the treatment of various types of cancer. In clinical trials, the drug is typically analyzed in combination with fulvestrant [50,51]. Results indicate that median progression-free survival was longer when fulvestrant was administered concurrently with sapanisertib. This data was collected from patients with advanced or metastatic ER+/HER2- breast cancer. However, increased toxicity was observed when these drugs were combined. The most common adverse events were nausea, vomiting, and hyperglycemia, which occurred more frequently with combination therapy. Clinical trials are also underway testing combination therapy combining fulvestrant with vistusertib. However, the results are unsatisfactory, as no significant improvement in outcomes, such as an extension of progression-free survival, has been observed [52,53].

#### TNBC Drugs

For TNBC, the following drugs are used: Samotolisib and Ipatasertib.

Samotolisib is a potent dual PI3K/mTOR inhibitor. When combined with prexasertib, it inhibits cell proliferation in TNBC cell lines [54]. It’s worth noting that no dose-limiting toxicity was observed, but the studies were not free of treatment-related adverse events. Leukopenia, thrombocytopenia, and nausea were common. Despite the challenges, the results are satisfactory. While the combination of the two drugs was associated with toxicity, synergistic antitumor activity was observed. Ipatasertib is a drug used in clinical trials for the treatment of cancer, tumors, solid tumors, breast cancer, and gastric cancer, among others. In TNBC, this drug was used in combination with paclitaxel compared to placebo [55,56,57,58]. The results were satisfactory, as the median progression-free survival was 1.3 months longer than placebo in patients. However, the studies were not free of adverse events. Diarrhea and neutropenia were relatively common. Although these side effects were bothersome and common, these are the first results confirming the effectiveness of targeted therapy in TNBC. The use of ipatasertib in patients diagnosed with TNBC requires further research.

Is it possible to clearly and easily compare therapies and drugs targeting the PI3K vs. AKT pathways? Each drug is different. Although the responses are positive, they are almost always associated with toxicity, causing side effects to a greater or lesser extent. When considering treatment and drug selection, it is worth analyzing already registered clinical trials and considering the potential benefits and side effects of the proposed therapy.

Resistance to PI3K/AKT/mTOR pathway inhibitors results from both inherited and acquired mutations that restore the activity of this pathway. Biomarker-based patient stratification utilizes genetic and protein markers, such as PI3K and AKT mutations, elevated phosphorylated protein levels, and cancer stem cell markers, to identify individuals most likely to respond to specific therapies. This approach allows for increased treatment efficacy and supports the selection of therapeutic strategies, including combination therapies. Cancer tumors can activate parallel or bypassing signaling pathways, compensating for inhibition of the PI3K/AKT/mTOR axis. Furthermore, blocking this pathway can suppress protective autophagy, which may contribute to the development of resistance. Identifying patients predisposed to respond to PI3K/AKT/mTOR inhibitors is crucial for optimizing treatment. Biomarker profiling enables precise patient selection for both monotherapy and combination therapies to overcome resistance. Biomarkers can predict which patients are most likely to respond to treatment and identify those at risk of developing resistance, allowing for proactive adaptation of treatment strategies [59,60,61].

### 2.2. Anti-HER2 Therapy

In the case of HER2-positive tumors, the first-line treatment is humanized anti-HER2 monoclonal antibodies (mAbs): trastuzumab (Herceptin), introduced in 1998, and pertuzumab (Perjeta), introduced in 2012 as one of the first immunotherapies approved for the treatment of breast cancer [62]. They are designed to bind to the receptor, preventing its activation, while limiting HER2 overexpression, which inhibits the intracellular signaling pathway. Trastuzumab has been shown to inhibit cancer cell proliferation by promoting cell cycle arrest in the G1 phase, but does not affect receptor dimerization, while pertuzumab additionally inhibits heterodimerization with other ERBB family proteins (mainly HER3). In addition to the direct anti-cancer effect, indirect mechanisms are also activated: through the engagement of Fcγ receptors (FCGR), antibody-dependent cellular cytotoxicity (ADCC) or antibody-dependent cellular phagocytosis (ADCP) is stimulated, a strategy that has been recognized in recent years as key to the synergistic mechanism of action (MAO) [63]. Additionally, the indirect effect of mAbs is achieved by complement activation via the classical pathway—complement-dependent cytotoxicity (CDC) and complement-dependent phagocytosis of cancer cells (CDCP) occur [64]. Due to differences in the mechanism of action (taking into account the differences in the HER2 epitopes with which both antibodies bind) and the low efficacy demonstrated in clinical studies in monotherapy in order to maximize the therapeutic effect (enhanced antitumor activity, also against disseminated tumors), these two drugs are usually used simultaneously, especially in the case of an insufficient response in monotherapy with trastuzumab, although the basis for the increased efficacy of this combination has not yet been fully explained [65,66]. In addition, in recent years, many researchers have drawn attention to the use of so-called antibody–drug conjugates (ADCs), which, in addition to mAb, additionally contain cytostatics active against a given type of cancer cells, and thanks to the precise internalization of monoclonal antibodies, they can be delivered directly to the patient’s cells, thus minimizing the side effects of systemic toxicity of classic chemotherapy [67]. By using such a combination, the amount of the drug (its concentration) that reaches the tumor directly is increased. Focusing on this drug delivery model, biomolecular ADC complexes combining trastuzumab with cytotoxic drugs targeting microtubule inhibition have been developed: Ado–Trastuzumab emtansine (T–DM1; Kadcyla; registered in 2013) and topoisomerase inhibition: Fam–Trastuzumab deruxtecan (T–DXd; Enhertu registered in 2020). Ghanashyam Biswas et al. have proven that TDM–1 is more effective than trastuzumab in preventing relapses in the long term, prolongs progression-free survival (PFS) and improves survival rates in patients with HER2(+) breast cancer, while preclinical studies are promising, in which T–Dxd shows activity against tumors resistant to T–DM1 [68,69,70,71].

Tyrosine kinase inhibitors (TKIs) targeting the intracellular domain of the HER2 receptor have also been used in HER2-positive breast cancer. By competing with ATP, they limit the phosphorylation of tyrosine residues, which inhibits signaling to the cell nucleus [72]. TKIs block enzymatic activity, which prevents excessive activation of the PI3K/Akt and MAPK pathways that determine cancer cell survival and proliferation [73]. In HER2 (+) breast cancer, they have been registered for about a decade and include lapatinib [74](Tykerb; 2007), neratinib [75], pyrotinib [74] , and tucatinib [76] , while afatinib has not yet been registered for this indication. Lapatinib is a dual inhibitor, inhibiting both EGFR and HER2. Its efficacy has been demonstrated mainly in mBC in combination with chemotherapy (it extended overall survival from 20.5 months for paclitaxel in monotherapy to 27.8 months in combination therapy) [74]. In the case of triple-positive BC (HR+, HER2+) it is used in combination with AI (letrozole). Similarly to tucatinib, it is an inhibitor with reversible action, unlike neratinib, pyrotinib and afatinib, which bind to the target receptor in an irreversible manner. TKIs are drugs with proven good synergistic effect, therefore they often appear in combination treatment regimens to increase the efficacy of therapy. Studies on further therapeutic combinations and assessment of their efficacy are also conducted [75,76].

### 2.3. Immunotherapy

Immunotherapy uses the patient’s own immune system and, in addition to the aforementioned monoclonal antibodies in the treatment of HER2-positive cancer, has found its application to an even wider extent in the case of TNBC therapy, because here it has the potential to significantly affect the quality of life of patients by reducing the share of classic chemotherapy [77]. In addition to the aforementioned mAbs targeting the HER2 receptor, checkpoint inhibitors (ICIs) are used here. These are PD-1 and PDL-1 (Programmed cell death—protein and ligand 1) inhibitors, because cancer cells, in order to avoid recognition and destruction by the T lymphocytes of the patient’s immune system, have developed an adaptive mechanism in which the ligand expressed on their surface binds to the PD-1 receptor on the cells of the immune system and in this way, mimicking physiological mechanisms, inhibits the proliferation of T lymphocytes and promotes their depletion—it silences the immune response, which allows the tumor to escape from the host’s own mechanisms [78]. In recent years, PARP inhibitors have become a breakthrough in the treatment of TNBC, particularly in patients with BRCA1/2 mutations. Their mechanism of action is based on the concept of synthetic lethality, in which cancer cells deficient in homologous recombination repair are selectively attacked, leading to their death. According to Jie et al., clinical trials have demonstrated the effectiveness of PARP inhibitors, such as olaparib and talazoparib, in improving progression-free survival in patients with germline BRCA mutations. Currently, there are projects aimed at using PARP inhibitors in TNBC without BRCA mutations. Another example of future research is the use of inhibitors in combination with other therapies, such as chemotherapy. The inclusion of PARP inhibitors in TNBC therapy requires further research, particularly clinical trials [79].

The main principle of action of PARP inhibitors is to block the repair of single-strand breaks in DNA, leading to the accumulation of double-strand breaks that are lethal to cells with defective HRR mechanisms, such as cells with BRCA mutations [80]. Five PARP inhibitors have been developed to date: olaparib, niraparib, rucaparib, talazoparib, and veliparib. These drugs differ in their ability to bind PARP to DNA and in their ability to inhibit its catalytic activity. They have demonstrated efficacy in the treatment of triple-negative breast cancer (TNBC) in patients with BRCA mutations, exacerbating DNA damage and leading to the death of cancer cells with defective DNA repair mechanisms. TNBC is a particularly aggressive subtype of breast cancer, affecting 10–15% of patients [81]. Compared to other types of cancer, TNBC has a three-fold higher risk of recurrence. It is characterized by the absence of estrogen, progesterone, and HER2 receptors, which for years limited treatment options to chemotherapy alone. However, new therapies are now available, such as immunotherapy (e.g., pembrolizumab), PARP inhibitors, and novel antibody–drug conjugates (e.g., sacituzumab govitecan), which have significantly improved the prognosis for patients with metastatic TNBC [82]. Immunotherapy has proven particularly important because, unlike other subtypes of breast cancer, TNBC lacks “classical” therapeutic targets. This treatment is usually better tolerated than chemotherapy, although it can lead to immune-related side effects. In most cases, they can be controlled with corticosteroids, but more serious toxicity develops in approximately 14% of patients, requiring careful consideration of the benefits and risks, especially when treated early in the disease course [82]. In the last year alone, 10 clinical trials were registered that evaluated the effectiveness of immunotherapy in breast cancer. Table 1 presents 3 clinical trials registered in the last year (2024–2025) and already underway.

It is worth noting that each combination therapy and radiotherapy has a molecular effect on cells. Current molecular combination therapy can target specific targets based on the patient’s tumor characteristics [83]. To optimize therapeutic outcomes and minimize the likelihood of recurrence after surgery, some combination therapies are currently used as adjuvant or first-line treatment for recurrent or metastatic breast cancer in clinical settings [84]. Due to the specificity of new and precise combination therapies, side effects can often be minimized, for example, by altering the therapeutic approach throughout treatment, or by changing the drug regimen, known as combination therapy. With any combination, there are risks to treatment effectiveness due to the use of multiple drugs. This can lead to problems such as inflammation, renal or liver failure, thrombocytopenia, and brain metastases. As with any new treatment, the potential for adverse effects and toxicity must be considered for each combination. A promising potential solution to this problem is the use of molecular docking system to aid in the development of the most effective combination treatment regimens. However, an increase in the number of extended clinical trials will improve the efficacy of therapy for patients with various subtypes of breast cancer [85]. Radiotherapy is increasingly used in combination therapies, supported by clinical trials. Radiotherapy damages cancer cells by destroying their DNA. This can occur directly, through ionizing radiation, or indirectly, when radiation stimulates nearby molecules that produce free radicals. These free radicals create reactive oxygen species (ROS), which stress and damage the cell. Cancer cells sometimes defend themselves by activating survival mechanisms, which can allow tumor recurrence. How well a tumor responds to treatment depends on its radiosensitivity (ease of destruction) or radioresistance (resistance to damage). Radiation alters the way cancer cells grow and divide, engaging them in cell death processes such as apoptosis. Depending on the patient’s condition, different treatment regimens are selected to achieve the best possible outcome [86].

### 2.4. TNBC Cancer Therapy

The exceptional genomic heterogeneity of TNBC, including its molecular subtypes, along with the lack of expression of hormone receptors and HER2, limits the possibilities of targeted therapy, as none of the hormonal therapy options or monoclonal antibodies used in other cancers show efficacy in this case [87]. So far, the basic treatment regimen has been mastectomy or sparing surgery (depending on the stage of the cancer), radiotherapy and neoadjuvant or adjuvant chemotherapy, which has its many limitations. The main aspect is the intrinsic or acquired resistance to chemotherapeutic agents, which translates into a high relapse rate. Focusing on the molecular stratification of TNBC subtypes could bring benefits in the field of targeted treatment and the development of immunotherapy. A conventional chemotherapy regimen includes the following options: Anthracyclines (doxorubicin, epirubicin), Regimens with anthracycline and cyclophosphamide:AC: doxorubicin and cyclophosphamide and ACT: doxorubicin, cyclophosphamide, paclitaxel, Taxane regimens (paclitaxel, docetaxel), TC: docetaxel and cyclophosphamide, TFEC (docetaxel, 5-fluorouracil, epirubicin, cyclophosphamide), Other Schemes: CMF (cyclophosphamide, methotrexate, 5-fluorouracil), Antimetabolites (gemcitabine or capecitabine), microtubule-inhibiting drugs (vinorelbine), and Antitubulin drugs other than taxanes: eribulin, ixabepilone.

The choice of drug is individualized depending on the size of the tumor, the lymph node involvement status, the general performance status or the presence of comorbidities, but anthracyclines in combination with a taxane are most often used, as it was proven in a meta-analysis of 86 randomized studies on 100,000 patients that adding anthracyclines to the taxane therapy regimen reduced the risk of relapse by 14% and the annual mortality by 12% [88]. Adding any other drug is considered a new treatment regimen. Despite early good responses to this type of treatment, there is no positive correlation between early complete remission and overall survival. It has been proven that as many as 90% of failures in mBC treatment result from the development of multidrug resistance [89]. Cancer cells have developed various resistance mechanisms that allow them to maintain viability after exposure to chemotherapeutic agents. Understanding them can significantly affect the efficacy of therapy and avoid toxic side effects in systemic chemotherapy, as well as the development of new drugs that take into account changes in cancer cells. The most important of them are ABC transporters, overexpression of β-tubulin III, mutations in DNA repair enzymes, changes in genes responsible for apoptosis and signaling pathways ALDH1, GSH/GST (glutathione/glutathione-S-transferase), and NF-ĸB.

Over the past two years, the U.S. Food and Drug Administration (FDA) has approved several new drugs and therapeutic indications for the treatment of breast cancer (Table 2). The most important include inavolisib in combination with palbociclib and fulvestrant for advanced breast cancer [90]; datopotamab deruxtecan for patients with unresectable or metastatic hormone receptor-positive (HR+) and HER2-negative breast cancer [91]; and capivasertib in combination with fulvestrant for patients with mutations in the PIK3CA, AKT1, or PTEN genes in HR+/HER2- breast cancer [92].

Inavolisib (novel PI3K inhibitor): Approved for the treatment of advanced breast cancer, in combination with palbociclib and fulvestrant. Datopotamab deruxtecan (novel antibody–drug conjugate): Approved for the treatment of unresectable or metastatic HR+, HER2- breast cancer. Capivasertib (novel inhibitor): Approved in combination with fulvestrant for patients with HR-positive, HER2-negative breast cancer with a PIK3CA/AKT1/PTEN mutation [90,91,92].

The failure rate of currently used immunotherapies in unselected populations of patients with TNBC remains high, and response rates to checkpoint inhibitor monotherapy often do not exceed 10%. However, significant improvements are being observed with combination therapies, such as the combination of immunotherapy with chemotherapy, which is currently undergoing intensive clinical trials. Combining immunotherapy and chemotherapy can lead to synergistic antitumor effects, making this approach promising in the treatment of TNBC. The effectiveness of immunotherapy is strongly dependent on the expression of PD-L1—a biomarker present on both tumor cells and immune cells—which predicts potential treatment response. Using immunotherapy as a first-line therapy, especially in previously untreated patients, may yield better results than its application after multiple prior lines of therapy [93,94].

### 2.5. Limitations and Challenges

Compared to traditional cancer treatments such as surgery, radiotherapy, and chemotherapy, immunotherapy is significantly more effective in prolonging patient survival. This is due to its ability to target multiple stages and targets of tumor development, while selectively attacking tumor tissue and limiting damage to healthy cells. However, cancer cells can develop mechanisms that allow them to escape immune surveillance, promoting metastasis and relapse—two key causes of death in cancer patients. Immunotherapy utilizes a variety of strategies, including stimulating innate and adaptive immunity, overcoming immunosuppressive mechanisms, and strengthening the body’s existing defenses. As a result, immunotherapeutics not only enhance the immune response at the primary tumor site during treatment but also induce systemic and long-lasting protective effects, helping to prevent metastasis and tumor recurrence. To date, cancer immunotherapy has been used in the treatment of many advanced malignancies, but its development faces significant barriers. One of the main problems is toxicity—its occurrence and severity can limit further progress and widespread implementation of these methods, as confirmed by numerous clinical trials. Therefore, a key challenge remains developing ways to reduce the risk of adverse events. Another important issue in immuno-oncology is the variable patient response to treatment. While some patients demonstrate significant improvement, others do not respond to immunotherapy at all. Although this phenomenon was not observed in the analyzed studies, this problem is well documented and requires further research to improve the effectiveness of therapy in a broader group of patients [95].

Another significant problem is the lack of selective toxicity, which means that many therapies affect not only cancer cells but also healthy tissues. This imprecision not only leads to reduced therapeutic efficacy but also increases the risk of damage to healthy tissues. Consequently, lower drug doses are often necessary, further limiting treatment effectiveness. An additional challenge is the tendency of conventional therapies to accumulate in healthy tissues at levels significantly higher than within the tumor. This results in suboptimal drug concentrations in cancer cells, meaning they are insufficient to achieve the desired therapeutic effect. As a result, treatment effectiveness is further reduced [96].

## 3. Future Research Directions in Breast Cancer Treatment—Nanomedicine

Although each year new drugs are approved and used in the treatment of breast cancer, none are 100% effective or guaranteed to cure the cancer. Therefore, developing new methods and treatment plans can increase survival, inhibit tumor growth, and even prevent metastasis [97]. The use of nanoparticle-based systems has recently attracted considerable interest in breast cancer treatment [98]. Nanomedicine offers opportunities that can improve the efficacy of administered drugs, enable better tissue penetration of the therapeutic system, and make the tumor more sensitive to a specific form of treatment [99].

These specially designed molecules have found wide application in various fields of medicine, including oncology, both in terms of diagnostics, as contrast agents (in combination with fluorophores or radioisotopes), and treatment, as carriers for drugs (chemotherapeutics, siRNA, radiosensitizers, immunostimulants) [100]. Thanks to this, nanoparticles can play an important role in oncology by killing cells through various mechanisms: generating reactive oxygen species (ROS), down- and up-regulation in protein production pathways, immunological interactions, and inhibition of transcription [101]. Nanoparticles, due to their size (1–100 nm) and ability to overcome biological barriers, enable precise, intracellular drug delivery and controlled release, which ensures high bioavailability of therapeutic compounds, while limiting the doses of systemically used chemotherapeutics with their simultaneously increased local concentration within the tumor [102]. Their main advantages are the reduction in systemic toxicity of conventional chemotherapy, wide possibilities in creating nanoformulas depending on the needs and precise targeting of cancer cells, and their destruction using electrical, magnetic or optical properties. Additionally, they are bioresponsive structures that can respond to changes inside the body such as pH or temperature changes, which improves the controllability, selectivity and effectiveness of the therapy [103]. Depending on the desired properties, nanoparticles can be made of polymers, lipids, polysaccharides of metal proteins, metal oxides or carbon, which constitute the core of the molecule and functional groups—one or more. They can be polymers, dendrimers, or nanotubes or take liposomal or micellar forms [104]. Two main mechanisms of drug delivery by nanoparticles have been characterized: passive and active. Passive transport, using the specificity of the tumor microenvironment: new, dynamically emerging, defective and leaky blood vessels and lack of or limited lymphatic drainage determine the effect of increased permeability and retention (EPR) [105].

However, this is a heterogeneous phenomenon within different tumors and even within different regions of the same tumor, therefore multifunctional nanoparticles are used, which have the ability to escape from blood vessels independently of EPR, generating local hypertension, loosening intercellular connections of the endothelium or inducing new damage within the tumor blood vessels [106]. In active transport, nanoparticles use surface antigens specific for target cells, different for each type of tissue, which enables selective delivery of the drug to the tumor area [107]. The first doxorubicin-based nanoparticle was registered 30 years ago. To date, liposomes, polymers, micelles, and carbon nanotubes formulated with the doxorubicin nanoparticle have been developed. Combining the nanocomposite with the drug demonstrates superior results compared to standard treatment with the drug alone, for example, in mouse models [108]. The nanodiamond–doxorubicin complex increases apoptosis and inhibits tumor growth and lung metastasis of breast cancer [109]. Nanomedicine enables the broad development of anticancer therapy based not only on chemotherapeutics, but also on therapy directed at siRNA (small interfering RNA), which, taking part in the RNA interference process (RNAi), is able to silence the expression of selected oncogenes by compatible binding to specific mRNA and its degradation [110]. Such a specific treatment deprives the cancer cell of a number of proteins responsible for growth, metastasis, or generating mechanisms of resistance to the applied treatment. This is a promising field of research, but the siRNA delivery system is a challenge, as these are molecules that have their grip directly inside the cell [111].

Although the first siRNA-based drug was registered in 2018 for the treatment of hereditary transthyretin amyloidosis (hATTR), followed by three others for different indications, research on molecules targeting breast cancer is ongoing. Currently, the biggest challenge is to deliver siRNA to the tumor in vivo, because these molecules have a short half-life (about 10 min) and are poorly taken up by cells due to their high molecular weight and the anionic charge of the phosphodiester backbone [112]. They are also rapidly degraded by endonucleases contained in serum and quickly removed by glomerular filtration. Therefore, nanocarriers are used, which protect siRNA from undesirable interactions, metabolism or degradation and have a beneficial effect on the targeted delivery of molecules. Viral and non-viral vectors are used in siRNA delivery, which are becoming increasingly popular due to their potentially lower toxicity and elimination of insertional mutagenesis. Non-viral vectors include positively charged vectors such as cationic cell-penetrating peptides, cationic polymers, dendrimers, conjugation of siRNA with small molecules such as cholesterol or bile acids (bioconjugates) and lipid-based nanocarriers (micelles, liposomes, microemulsions and solid lipid nanoparticles) [113]. The use of nanoparticles enables the delivery of many thousands of siRNA molecules to the cell, while conjugates using antibodies only 1–10 molecules [114]. In addition, their size in the range of 30–200 nm potentially prolongs systemic clearance, because it is not filtered in the renal tubules but is not retained in the reticuloendothelial system of the liver and spleen (additional support in the construction of NC is the use of PEG). NCs are used that combine siRNA targeting a gene that determines the resistance mechanism with a chemotherapeutic agent, in order to be combined with deposition in the cell to obtain a synergistic cytotoxic effect [115]. NC containing siRNA, depending on the targeting of individual genes, may affect specific features of the tumor: metastasis—PTPN22 phosphatase, NF– ĸB p65 subunit, Twist transcription factor, VEGF, Lcn2 glycoprotein, PLK1 kinase, drug resistance: Pgp glycoprotein, multidrug resistance protein 1 (MRP– 1), autophagy-related protein 7 (ATG7), CXCR4 chemokine receptor, MTDH metadherin, HER2, immune evasion: VEGF, macrophage migration inhibitory factor (MIF), CCR2 receptor, PITPNM3 protein, PD– 1 or PD– L1 and CD73 [116].

### Nanoaqualip Technology

**Figure 4:**
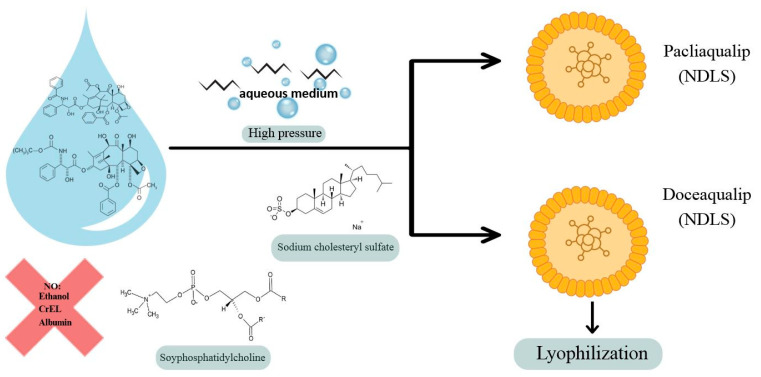
Nanoaqualip technology. The particles are uniform, approximately 100 nm in size, and are produced in an aqueous environment under high pressure using soy phosphatidylcholine and sodium cholesterol sulfate. This suspension is then lyophilized. Dilution is performed in 5% dextrose solution. Nanosomal lipid suspensions of paclitaxel (Pacliaqualip; NPLS) and docetaxel (Doceaqualip; NDLS) have been developed using this technology. The goal was to develop formulations with a superior safety profile to conventional taxanes. CrEL—Cremophor EL—formulation vehicle.

Particles developed using “NanoAqualip” technology can increase drug availability by improving permeability and retention in damaged tumor blood vessels. These particles eliminate the need for corticosteroid premedication due to the significantly lower risk of hypersensitivity compared to conventional drug administration (Figure 4). Application of these particles provides improved response rates and better tolerability in locally advanced or metastatic breast cancer compared to conventional drug administration. There is increasing evidence that this technology is effective and safe for local therapy in advanced breast cancer [117,118]. Another example of the use of nanomedicine in breast cancer treatment in recent years has been the so-called Nanoaqualip technology [119,120].

This technology involves the formation of a lipid-based nanomedicine in an aqueous environment. This eliminates the involvement of toxic drug solvents, primarily ethanol, preventing systemic toxicity and many other serious adverse effects. This eliminates the need for premedication with glucocorticosteroids (GCs) before drug administration [119].

Both NPLS and NDLS are currently undergoing clinical trials and demonstrate safety and high efficacy—pCR and CR response rates, as well as overall survival, remain comparable to the previously used formulation, docetaxel [117]. These drugs appear promising, particularly in patients with advanced or metastatic cancer, in patients who would prefer to avoid GCS, and in those who, for some reason, are at increased risk of hypersensitivity reactions.

Experiments using nanoparticles as a supportive therapy for breast cancer are already in advanced clinical trials. In the last five years alone, seven clinical trials have been officially registered in Phase II or Phase III on the ClinicalTrials.gov website. Table 3 presents ongoing clinical trials registered over the past five years.

A major challenge facing nanomedicine is the interaction of nanoparticles with biological systems and their long-term cytotoxicity, which is still rarely studied and reported in current reports. The impact of inorganic nanoparticles is of particular concern, as ions released upon their degradation can disrupt the metabolism and proliferation of cancer cells [121].

Furthermore, the Nanoaqualip technique faces significant translational challenges, including difficulties in scaling production due to the complexity of the nanomaterials, high manufacturing costs, unclear and evolving regulatory guidelines for new nanomedicines, and the need for rigorous analytical methods to ensure consistent characterization of nanomaterials for safety and efficacy. Nanomaterial production is inherently more complex than conventional drug formulations, which hinders the development of standardized large-scale processes. The lack of uniform production standards across the industry limits the ability to obtain reproducible, high-quality batches, and the need for costly and extensive safety testing further increases the costs and lengthens the approval process for nanomedicines such as Nanoaqualip [117,118,119,120].

Despite promising results in preclinical models, the clinical implementation of nanomedicines is slow and often challenging. General protocols are lacking, characterization of materials and biological mechanisms remains insufficient, and the statistical analyses used do not always ensure complete reliability of results. Furthermore, heterogeneity in research models, limited data sharing, and flaws in study design hinder the progression of nanomedicines to late-stage clinical trials.

Creating interdisciplinary teams that combine materials science, characterization of new technological platforms, and disease models that best reflect clinical conditions, while shaping a regulatory framework consistent with high scientific standards, will generate the data necessary for approval. This will enable the introduction of breakthrough technologies that address unmet needs in healthcare and cancer therapy [122].

Surgery, chemotherapy, and radiotherapy are the most frequently chosen treatment methods for primary tumors. Cancer recurrence, metastasis, and treatment failure necessitate the use of additional methods. Immune-targeted therapies can be a milestone in cancer treatment. Increasingly frequently conducted and registered preclinical studies suggest that the long-term success of cancer therapies depends on immunotherapy. Therefore, cancer immunotherapy is considered an effective treatment method that eliminates both primary tumors and metastases. Nanomedicine is an innovative platform enabling the simultaneous delivery of various immunomodulatory factors directly to the tumor environment or regional lymph nodes. Its primary goal is to precisely reprogram or modulate the immune response through targeted interventions on specific signaling pathways. The development of cancer immunotherapy, primarily using monoclonal antibodies and T-cell-based cell therapies, represents a breakthrough in oncology. However, small-molecule immunotherapeutics are gaining increasing interest. Compared to biologics, they exhibit favorable properties, such as a simpler chemical structure, better tissue penetration, lower production costs, higher stability, and longer shelf life. However, their rapid systemic distribution remains a limitation, potentially leading to off-target side effects. The use of nanotechnology in the formulation of these compounds opens the possibility of increasing their bioavailability and selectivity, facilitating precise targeting of specific immune cell subpopulations while reducing systemic toxicity. Nanoimmunotherapy is challenging, but these two approaches complement each other, improving the overall antitumor response rate [123,124].

It’s worth mentioning that, like other methods, this type of therapy has certain limitations. In the case of liposomes, these primarily concern stability and effective drug storage. Furthermore, there are often limitations to large-scale production. Furthermore, the short half-life can be problematic when it comes to delivering the drug over longer distances and its gradual release. Lipid nanoparticles have been reported to have side effects that can cause inflammation. Other limitations may include heterogeneous physicochemical properties, crystallization, low drug loading efficiency, emulsifier toxicity, and the potential cytotoxicity of some polymer components. To enable the further development of nanoparticles as promising tools in cancer therapy, the development of global regulatory standards is essential, requiring close collaboration between international regulatory agencies, academia, and industry. However, scaling up pharmaceutical nanoparticles presents a number of complex challenges. Their complex structure requires a thorough understanding to identify key properties necessary for reproducibility. This step provides the basis for selecting appropriate industrial-scale manufacturing processes and defining critical analytical parameters. Transitioning from basic research to commercial production of therapeutic nanoparticles requires resolving numerous challenges related to chemistry, manufacturing technology, and quality control. Proving the transferability of a given technology to a specialized development center or contract manufacturing organization, where scalable, cost-effective, and well-controlled processes can be implemented, is a key element. The complexity of the therapeutic nanoparticle manufacturing process further complicates the transition from preclinical trials to clinical trials and full commercialization. To meet Good Manufacturing Practice (GMP) requirements, it is recommended to use proven unit operations already used in the pharmaceutical industry, which can accelerate the process of implementing new therapies [125].

## 4. Conclusions

Breast cancer therapy is undergoing continuous improvement and research into increasingly precise therapeutic solutions. Drugs based on well-known hormonal mechanisms involved in the pathogenesis of this cancer are still being actively developed, but more and more drugs are being implemented that target molecular targets related to carcinogenesis, such as the PI3K/AKT/mTOR pathway. In addition, many current clinical trials compare the use of existing and registered drugs in various combined regimens, sometimes giving better treatment results. The implementation of nanoparticles as a drug carrier system has been groundbreaking, showing great potential to become the basis for diagnostics and treatment thanks to their unique properties ensuring higher efficacy, but also safety for patients. They can find a special place in TNBC therapy, especially in advanced and metastatic forms, where standard treatment regimens do not bring satisfactory results. They enable the transport of not only chemotherapeutics, but also siRNA, which translates into the precision and multidirectionality of the agents used. The number of clinical trials emerging each year indicates that new treatment methods are being sought. Combination therapies and synergistic approaches herald further attempts to increase the effectiveness of breast cancer treatment. This is a decisive and positive impulse, with hope for the future of oncology.

## Figures and Tables

**Table 1:** Registered clinical trials from the last year in which immunotherapy was selected in breast cancer treatment. Based on ClinicalTrials.gov.

Countries	Study Type	Phase	Official Title	Detailed Description	Interventions	Years and Status
Beijing, China	Interventional	Not Applicable	Safety Assessment of Concurrent Radiotherapy and Novel Systemic Therapy for Breast Cancer	The aim of the study is to evaluate the safety of combination therapy in patients with lymphatic drainage within the chest wall/breast requiring capecitabine, a CDK 4/6 inhibitor, HER2-targeted therapy, or immunotherapy.	Radiation HER2 inhibitors, CDK4/6 inhibitors, PARP inhibitors, ICIs	2024–2027
Jacksonville, FL, USA	Observational	Not Applicable	Observational Basket Trial to Collect Tissue to Train and Validate a Live Tumor Diagnostic Platform	This study is being done to collect tissue samples to test how accurately a tumor response platform, Elephas, can predict clinical response across multiple types of immunotherapies, chemoimmunotherapy and tumor types.	Patients will receive standard treatment with checkpoint inhibitors and undergo standard tumor assessment during screening and follow-up.	2024–2027
This study has locations in Greece	Observational	Not applicable	Mechanisms of Response and Resistance to Innovative Treatments in Patients With Locally Advanced or Metastatic Breast Cancer	The study aims to utilize modern methods such as DNA sequencing, analysis of circulating genetic material, digital imaging, and radiological analysis, as well as data from patient medical records. Based on this, a machine learning algorithm will be developed that will predict whether a patient will respond well to treatment or develop resistance, depending on the genetics and molecular profile of the tumor.	Patients receiving treatment with an Antibody–Drug conjugate (e.g., Trastuzumab–Deruxtecan, Sacituzumab–Govitecan) Patients receiving treatment with an immune checkpoint inhibitor (ICI) (e.g., pembrolizumab) Patients receiving treatment with a PARP inhibitor (e.g., Olaparib)	2024–2029

**Table 2:** New drugs and their indications approved by the FDA in breast cancer treatment in the last 2 years (as of early 2025).

Drugs	Structure	Characteristics	Indications
Capivasertib	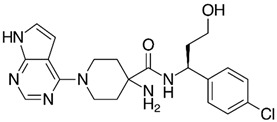	Capivasertib is a kinase inhibitor drug that works by blocking specific proteins that play a key role in the development and survival of cancer cells. This substance selectively inhibits the activity of the AKT protein (also known as protein kinase), a key component of the PI3K/AKT/mTOR signaling pathway. Overactivity of this pathway in cancer promotes uncontrolled cell growth and survival. Inhibition of AKT by capivasertib aims to limit or stop cancer cell proliferation.	Approved for the treatment of hormone receptor-positive, HER2-negative breast cancer with an abnormal PIK3CA gene
Datopotamab deruxtecan-dlnk	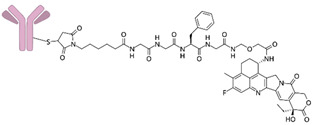	This drug binds to Trop-2 and internalizes the receptor, enabling transport of the active drug into the cell. The release of exatecan inhibits cell replication, leading to apoptosis. The process of exatecan penetration into neighboring cells triggers a series of processes that ultimately result in cell death.	Approved for the treatment of: hormone receptor-positive (HR+) and HER2-negative (HER2-) breast cancer. It is also being studied for the treatment of other types of cancer.
Inavolisib	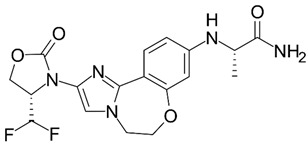	Inavolisib is a potent and selective inhibitor of the PI3K-alpha enzyme (phosphoinositide 3-kinase alpha). It is designed to inhibit the PI3K pathway through HER2-dependent degradation. It aims to inhibit tumor growth in patients whose cancers are caused by PI3K mutations.	Approved for the treatment of hormone receptor-positive, HER2-negative breast cancer with an abnormal PIK3CA gene. It is also being studied for the treatment of other types of cancer
Kisqali Femara Co-Pack (Ribociclib Succinate and Letrozole)	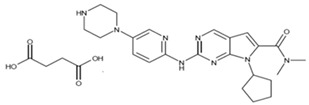 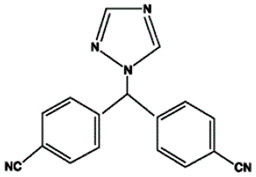	It inhibits the growth of cancer cells, which are ultimately destroyed.	Approved for the treatment of hormone receptor-positive (HR+) and HER2-negative (HER2-) breast cancer. Used for stage II or III breast cancer.

**Table 3:** Registered clinical trials from the last 5 years (2020–2025) in which nanoparticles were included in breast cancer treatment. Based on ClinicalTrials.gov.

Countries	Study Type	Phase	Official Title	Detailed Description	Interventions	Years and Status
Shanghai, China	Interventional	2	A Phase II Study to Explore the Safety, Tolerability, and Preliminary Antitumor Activity of Sitravatinib Plus Tislelizumab or Combination With Nab-paclitaxel in Patients With Locally Recurrent or Metastatic Triple Negative Breast Cancer (TNBC)	The aim of the study is to evaluate the efficacy of sitravatinib in combination with tislelizumab and nab-paclitaxel in patients with previously untreated metastatic breast cancer (TNBC) or with recurrence/metastasis after surgery.	Drug: Sitravatinib Drug: Tislelizumab Drug: Nab-paclitaxel	2021–2024
**Gothenburg, Sweden**	Interventional	2	Sentinel Lymph Node Localisation With an Ultra-low Dose of Superparamagnetic Iron Oxide Nanoparticles in Patients With Breast Cancer	The main objective is to demonstrate that the use of superparamagnetic iron oxide nanoparticles (SPIO) as a tracer is equally effective in detecting sentinel lymph nodes (SLNs) in breast cancer patients.	Drug: Superparamagnetic Iron Oxide Device: Technetium99	2023–2027
This study has locations in USA, Hong Kong, and Sweden	Interventional	3	Sentinel Lymph Node Biopsy in Ductal Cancer in Situ or Unclear Lesions of the Breast and How to Not do it. An Open-label, Phase 3, Randomised Controlled Trial. (SentiNot 2.0).	The aim of this study is to investigate the use of superparamagnetic iron oxide nanoparticles as a marker for delayed sentinel lymph node dissection in patients for whom initial axillary surgery is considered oncologically unnecessary and should be avoided. This includes patients with a preoperative diagnosis of ductal carcinoma in situ.	Diagnostic Test: Delayed SLND Diagnostic Test: Late SLND	2020–2027
Houston, TX, USA	Interventional	2	A Phase-2, Two-Cohort Trial of Neoadjuvant Nab-Paclitaxel and Alpelisib in Anthracycline Refractory Triple Negative Breast Cancer With PIK3CA or PTEN Alterations	The aim of the study is to evaluate the efficacy of nab-paclitaxel and alpelisib in the treatment of patients with triple-negative breast cancer with mutations in the PIK3CA or PTEN gene that does not respond to anthracycline chemotherapy (anthracycline-resistant).	Drug: Alpelisib Drug: Nab-paclitaxel	2020–2025
Gothenburg, Sweden	Interventional	1, 2	Sentinel Node Localization and Staging with Low Dose Superparamagnetic Iron Oxide-enhanced Magnetic Resonance Imaging and Magnetic Probe in Patients with Breast Cancer	The aim of this study was to determine whether detection of sentinel node status using ultralow dose superparamagnetic iron oxide nanoparticles is feasible.	Drug: Superparamagnetic Iron Oxide	2021–2024
This study has 30 locations in USA	Interventional	2	Randomized Phase 2 Clinical Trial of Nab-Paclitaxel + Durvalumab (MEDI4736) + Tremelimumab + Neoantigen Vaccine Vs. Nab-Paclitaxel + Durvalumab (MEDI4736) + Tremelimumab in Patients With Metastatic Triple Negative Breast Cancer	The Phase II study is examining the efficacy of the drugs nab-paclitaxel, durvalumab, and tremelimumab, in combination with or without a personalized synthetic long-peptide vaccine (neoantigen vaccine), in the treatment of patients with metastatic triple-negative breast cancer.	Procedure: Biopsy Procedure Procedure: Biospecimen Collection Drug: Carboplatin Procedure: Computed Tomography Biological: Durvalumab Drug: Gemcitabine Hydrochloride Procedure: Magnetic Resonance Imaging Drug: Nab-paclitaxel Biological: Personalized Synthetic Long Peptide Vaccine Drug: Poly ICLC Biological: Sacituzumab Govitecan Biological: Tremelimumab	2021–2025
This study has 1008 locations in USA and Puerto Rico	Interventional	2	(CompassHER2-pCR): Preoperative THP and Postoperative HP in Patients Who Achieve a Pathologic Complete Response	The aim of the study is to evaluate the efficacy of paclitaxel, trastuzumab, and pertuzumab in eliminating further chemotherapy after surgery in patients with HER2-positive stage II-IIIa breast cancer who, after preoperative chemotherapy and HER2-targeted therapy, had no cancer detected at the time of surgery (neither in the breast nor in the axillary lymph nodes).	Drug: Docetaxel Procedure: Lumpectomy Procedure: Mastectomy Drug: Nab-paclitaxel Drug: Paclitaxel Biological: Pertuzumab Radiation: Radiation Therapy Biological: Trastuzumab Biological: Trastuzumab Emtansine	2020–2038

## Data Availability

Not applicable.
